# Supplementation with Combined *Lactobacillus helveticus* R0052 and *Bifidobacterium longum* R0175 Across Development Reveals Sex Differences in Physiological and Behavioural Effects of Western Diet in Long–Evans Rats

**DOI:** 10.3390/microorganisms8101527

**Published:** 2020-10-05

**Authors:** Elizabeth M. Myles, M. Elizabeth O’Leary, Rylan Smith, Chad W. MacPherson, Alexandra Oprea, Emma H. Melanson, Thomas A. Tompkins, Tara S. Perrot

**Affiliations:** 1 Department of Psychology and Neuroscience, Dalhousie University, 1355 Oxford St., Halifax, NS B3H 4R2, Canada; elizabeth.oleary@dal.ca (M.E.O.); rylan.smith@dal.ca (R.S.); alex.oprea@dal.ca (A.O.); emma.melanson@dal.ca (E.H.M.); 2 Rosell® Institute for Microbiome and Probiotics, 6100 Ave. Royalmount, Montreal, QC H4P 2R2, Canada; cmacpherson@lallemand.com (C.W.M.); ttompkins@lallemand.com (T.A.T.); 3 Brain Repair Centre, Dalhousie University, Halifax, NS B3H 4R2, Canada

**Keywords:** probiotics, food intake, leptin, Western diet, anxiety, inflammation, sex differences, animal models, *Lactobacillus*, *Bifidobacterium*

## Abstract

The gut microbiome affects various physiological and psychological processes in animals and humans, and environmental influences profoundly impact its composition. Disorders such as anxiety, obesity, and inflammation have been associated with certain microbiome compositions, which may be modulated in early life. In 62 Long–Evans rats, we characterised the effects of lifelong *Bifidobacterium longum* R0175 and *Lactobacillus helveticus* R0052 administration—along with Western diet exposure—on later anxiety, metabolic consequences, and inflammation. We found that the probiotic formulation altered specific anxiety-like behaviours in adulthood. We further show distinct sex differences in metabolic measures. In females, probiotic treatment increased calorie intake and leptin levels without affecting body weight. In males, the probiotic seemed to mitigate the effects of Western diet on adult weight gain and calorie intake, without altering leptin levels. The greatest inflammatory response was seen in male, Western-diet-exposed, and probiotic-treated rats, which may be related to levels of specific steroid hormones in these groups. These results suggest that early-life probiotic supplementation and diet exposure can have particular implications on adult health in a sex-dependent manner, and highlight the need for further studies to examine the health outcomes of probiotic treatment in both sexes.

## 1. Introduction

The microbes that inhabit the animal gut (i.e., gut microbiota) have been consistently reported to affect physiological and psychological functioning [1]. These microbes—especially bacteria—have important physiological functions in the host, including helping to regulate digestion, the immune system, central nervous system, and hormone levels (see [2] for a review). Indeed, the composition of the microbiota has been linked to various diseases (see [3] for a review) and is highly vulnerable to environmental exposures of the host (e.g., antibiotic use, infection) [4]. These environmental experiences can alter the microbiome in a way that promotes disease development (i.e., dysbiosis) [4]. Importantly, dysbiosis can be compounded by unhealthy Western diet (i.e., a diet containing processed foods, refined grains, fats, and added sugar) [5], which has been shown to alter the microbiota composition and change how the host stores and uses energy [6,7].

Probiotics (e.g., bacteria, yeasts) are defined as “live microorganisms that, when administered in adequate amounts, confer a health benefit on the host” [8] (p. 507). Expanding on this definition, Dinan and colleagues [9] describe that certain probiotics can function as psychobiotics if the health benefit from administration is that there is an improvement of symptoms of psychiatric illness. Although the microbiota is a potential target for the treatment and prevention of specific diseases that may be related to dysbiosis (e.g., stress-related disorders, gastrointestinal disorders) [2,10], multiple groups have stressed that research has not sufficiently differentiated a “normal” or “healthy” microbiota from that which is “abnormal” or “unhealthy” [2,3,11]. Many of the existing studies that administer or report on specific probiotics emphasise that the effects of probiotic treatment are dependent on which microorganisms were used [12,13,14], making studies that aim to characterise specific strain or formulation effects critical for the field [8]. Indeed, specific probiotic formulations have been shown to decrease the response to stress in rodent models (e.g., *Companilactobacillus farciminis* [15]; CEREBIOME^®^ [16]; Lacidofil^®^ [17]), improve anxiety symptomatology in humans and anxiety-like behaviours in rats (e.g., CEREBIOME^®^ [18]), improve obesogenic outcomes (e.g., *Lactiplantibacillus plantarum* [19]; *Bifidobacterium* spp. [20,21]; *L. helveticus* R0052 [22]), and reduce the inflammatory response (e.g., *B. infantis* 35624 [23]; 8-strain combination probiotic [24]; 3-strain combination probiotic [25]).

Above and beyond the findings that probiotic treatment seems to ameliorate the previously mentioned health outcomes is the apparent interaction between the development of psychiatric disorders, obesity-related disorders, and inflammatory conditions. In their review, Dallman et al. [26] concluded that increased glucocorticoids and insulin work in conjunction to increase the consumption of palatable foods and energy storage as abdominal fat. Specifically, Pecoraro et al. [27] report reduced hypothalamic–pituitary–adrenal (HPA) activity (i.e., lower corticotropin-releasing factor (CRF) mRNA) when rats consume palatable food. Importantly, diet quality can also impair the development of the stress response in offspring mice, even without direct administration of the poor diet [28]. Psychological stress and dysfunction are also associated with increased inflammatory responses in both animal models [29,30] and human subjects [31] (see [32] for review on inflammation and anxiety disorders). In animal models, pro-inflammatory markers are increased in rats exposed to a high-fat diet (e.g., IL-6, TNF-α, IL-1β) [33]. In their review, Dandona et al. [34] report that obesity from overeating in humans may promote oxidative stress and inflammation. Of note is the association between obesity and type 2 diabetes and increased IL-6, TNF-α, and C-reactive protein (CRP) levels. Overall, probiotics that induce the production of IL-10 are described as immunoregulatory, which can improve health (e.g., reducing allergies, symptoms of irritable bowel disease) by inhibiting pro-inflammatory cytokines that induce inflammation [35]. Probiotics can also be categorised as immunostimulatory, where they have the potential to act against cancer and infection (e.g., inducing IL-12 production can induce natural killer cells) [35].

This study was designed to examine the effects of lifelong supplementation with CEREBIOME^®^ (i.e., *Lactobacillus helveticus* R0052 and *Bifidobacterium longum* R0175; Lallemand Health Solutions Inc., Montreal, QC, Canada) in Long–Evans rats of both sexes exposed to a control or Western diet on adulthood anxiety-like behaviours, obesogenic outcomes, and inflammatory responses to acute stress. Based on previous literature characterising the effects of the strains composing CEREBIOME^®^ or similar strains, we hypothesised that the probiotic formulation would be protective with respect to our measured adult health outcomes in comparison to a placebo. We additionally hypothesised that the Western diet would worsen these measured health outcomes compared to a control diet, and that probiotic administration may counteract some of the detrimental health consequences of lifelong Western diet intake.

## 2. Materials and Methods

### 2.1. Animal and Breeding

**Figure 1:**
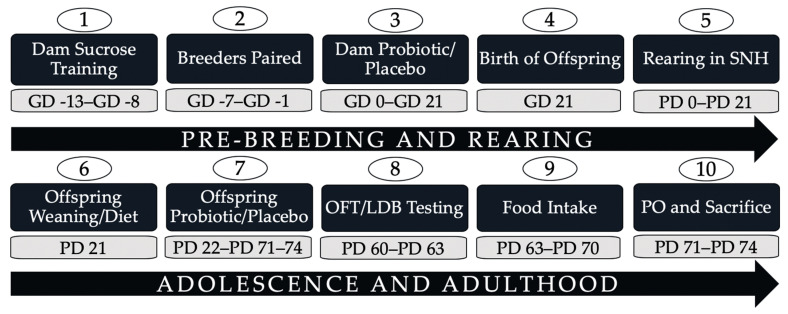
Experimental timeline (numbered in chronological order from 1 to10) from arrival of breeder rats until offspring sacrifices with gestational day (GD) 0 serving as the reference point for the timeline. PD: postnatal day (0 to 74); GD: gestational day (−13 to 21); SNH: semi-naturalistic housing; OFT: open field test; LDB: light–dark box; PO: predator odour.

The timeline for all experimental procedures is summarized in Figure 1. All procedures were approved by the Dalhousie University Committee on Laboratory Animals (Dalhousie University, Halifax, NS, Canada, protocol #18-023, 1 April 2018) and performed in accordance with the guidelines of the Canadian Council on Animal Care (CCAC). For this experiment, 16 Long–Evans hooded rats (eight males between 275 and 300 g and eight females between 220 and 225 g; Charles River Laboratories, Raleigh, NC, USA) were ordered—specific-pathogen-free and viral-antibody-free—for breeding and given two weeks to acclimate to our facility upon arrival. Rats were housed in same-sex pairs from arrival until breeding in polypropylene cages (47 cm × 24 cm × 20.5 cm) in a colony room maintained at 21 °C ± 2 °C under a reversed 12:12 h light–dark cycle with lights off at 10:00 in the multi-species and restricted card access animal facility in the Department of Psychology and Neuroscience (Dalhousie University, Halifax, NS, Canada). These standard static conventional housing cages were covered with stainless-steel wire hoppers and micro-isolator lids and contained softwood bedding (Shaw Resources, Shubenacadie, NS, Canada) with a black PVC tube (12 cm long, 9 cm in diameter). Standard rodent chow (Laboratory Rodent Diet 5001, Purina LabDiet^®^, St. Louis, MO, USA) and double-filtered municipal tap water were supplied ad libitum in a glass bottle with a stainless-steel sipper tip.

**Figure 2:**
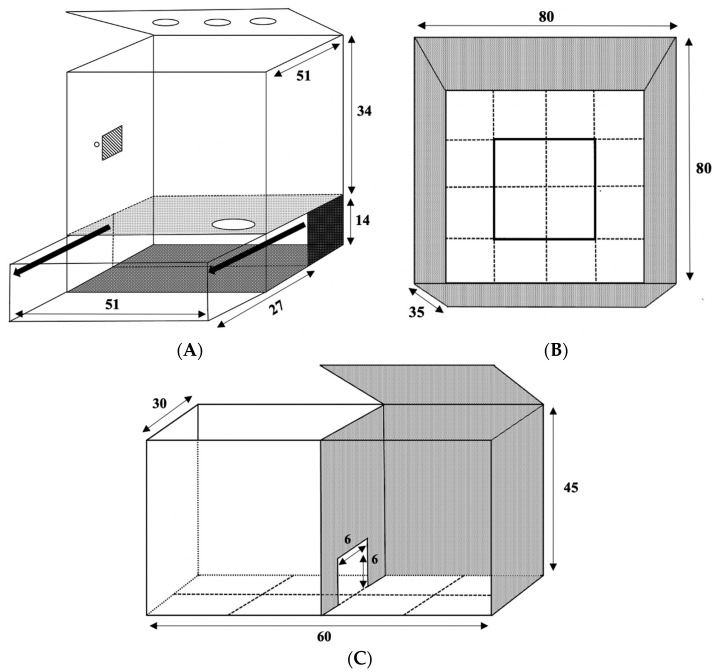
Visual representation (in centimetres) of (**A**) the semi-naturalistic housing cages with a burrow section that pulls open used in this experiment for offspring and their dam during lactation (PD 0 to PD 21), (**B**) The open field test, and (**C**) The light–dark box and predator odour exposure arena.

For breeding, two groups, each containing four naïve breeding pairs, were placed in separate colony rooms that would eventually become the probiotic or placebo administration rooms. The colony rooms were virtually identical in appearance and setup. Breeding in both rooms was conducted at the same time, and involved placing each of the eight pairs of rats together in a standard housing cage for seven days. After this time, the female was assumed to be pregnant, and the male was removed from the cage and housed alone until sacrifice. Females were pair-housed after breeding to minimise isolation stress until estimated gestational day 17 when they were given a fresh cage to await parturition. Females were weighed bi-weekly during pregnancy, and seven of eight bred females gave birth after 21 gestational days, with one female in the placebo-designated room not producing a litter. Within 24 h of parturition, females and their litters were transferred into large cages with a burrow section (semi-naturalistic housing, SNH, Figure 2A) [36]. Female breeders were kept with their pups until weaning at postnatal day (PD) 21.

A total of 46 pups were live-born (1 still-born) to four probiotic-treated dams with an additional 4 pups not surviving to weaning. In the placebo group, 33 pups were live-born (2 still-born) to three dams with an additional 3 pups not surviving to weaning. Due to the discrepancy in sample sizes (i.e., 42 probiotic offspring, 30 placebo offspring), pups in the probiotic group were culled to 32 offspring (i.e., 10 culled and used for molecular assay optimisation), which left 8 pups in each diet condition with an equal sex ratio. Overall, for this experiment, breeding produced 62 offspring for eight experimental offspring groups separated by treatment (32 probiotic, 30 placebo), by diet condition (31 Western diet, 31 control diet), and by sex (30 males, 32 females). Offspring were housed in same-sex pairs in groups of two or three in standard housing cages as described. Ethically, to reduce the number of offspring produced for this experiment, eight dams were bred, and offspring were sorted into the eight experimental groups randomly but in a way that maximised diversity. Due to litter sex ratios and ethical responsibility to pair house or house in threes, all litters could not always be represented in each group, but complete litter statistics are summarised in Table 1. Sample size was chosen from previous similar experiments by our laboratory group [36] and kept as small as possible to reduce the number of animals bred for experimental purposes.

### 2.2. Probiotic Administration

In this experiment, each dam and her offspring were placed into the same treatment group and received probiotic (CEREBIOME^®^) or its placebo. For all animals, the daily probiotic dosage was 1 billion colony forming units (CFUs) per mL, per day (i.e., 0.05 g of probiotic powder per 0.5 mL of reverse osmosis water, based on the CFU/g value for this specific batch of probiotic). An equivalent amount of placebo (i.e., the probiotic vehicle that contains malic acid, xylitol, and maltodextrin as excipients) solution was prepared (i.e., 0.05 g of placebo powder per 0.5 mL of reverse osmosis water). Solutions were made fresh each morning and maintained at 4 °C ± 4 °C until administration, according to the protocol developed in [37]. For both dams and offspring, probiotic or placebo were delivered via syringe feeding during the dark phase of the light–dark cycle (i.e., 11:00 ± 1 h) as per the protocol developed by Tillmann and Wegener [38] for adult male rats. Briefly, the syringe feeding protocol involves a training period that varies by age and sex of the rat, whereby the rat is lap-fed by an experimenter. After successful training (three to four days for adult male rats), rats will voluntarily approach the syringe and feed from the cage.

There is evidence suggesting that probiotics are transferred through the act of breastfeeding [39,40], and there are further reports that infant microbial gut colonisation may begin in utero [41]. Thus, although this experiment focused on offspring outcomes, solutions were delivered daily to the dam during pregnancy and lactation to maximise any potential benefits for the offspring. To expedite syringe training with the female breeders and to not interfere with breeding, females were trained by adding a 0.25 M sucrose solution (i.e., 0.04 g of sucrose in 0.5 mL of reverse osmosis water) to the solution for four days prior to breeding. Four of the eight females voluntarily took the syringe with sucrose-sweetened probiotic or placebo solution after the four days. After breeding, probiotic and placebo solution were delivered without added sucrose, and all females voluntarily fed from the cage at that time. Solutions were delivered to the offspring daily from PD 22 until day of sacrifice (PD 71 to 74; i.e., for equal to or greater than seven weeks). As the probiotic vehicle contained a polysaccharide food additive that was slightly sweet, sucrose was not added to the offspring probiotic and placebo solutions during training. All offspring learned to take the solution voluntarily from the cage after 27 days.

### 2.3. Diet Manipulation

From weaning, all rats were fed a specific chow and provided water ad libitum until sacrifice. Diets were purchased from Research Diets (New Brunswick, NJ, USA) and comprised equivalent protein matched by the supplier and a similar number of kilocalories. Offspring rats in both the probiotic and placebo groups received either control diet (product #D14042701; carbohydrate 73% kcal, fat 10% kcal, protein 17% kcal, kcal/g = 3.9) or Western diet (product #D12079B; carbohydrate 43% kcal, fat 40% kcal, protein 17% kcal, kcal/g = 4.7). In the control diet, the main sources of carbohydrates were corn starch followed by maltodextrin, whereas they were sucrose, maltodextrin, and corn starch in the Western diet. The main source of fat for both diets was anhydrous milk fat, but the Western diet contained more anhydrous milk fat relative to the control diet; they contained equal amounts of corn oil. Protein content, vitamins, minerals, and added fibre were identical between the two diets, but the Western diet contained cholesterol, whereas the control diet did not.

### 2.4. Weight and Food Intake

Offspring rats were weighed bi-weekly from weaning until sacrifice to monitor for any changes in health status and also at an average age of PD 67 to obtain a final adult weight. All birth, wean, and adult weights are provided in grams (g). Additionally, when offspring reached adulthood, food intake was measured for five consecutive days between PD 63 and PD 70. Briefly, a 24 h ± 1 h change in food weight for each cage was determined and divided by the number of rats in the cage. A 4-day average amount of food consumed (in kcal, by multiplying each average food weight in grams by the kcal/g values above) was calculated from the change in food across each of the five measurement days, and differences by treatment, diet, or sex were determined.

### 2.5. Anxiety-Like Behaviours

To assess anxiety-like behaviour, all offspring were tested for 5 min in each of the open field test (OFT; between PD 60 and PD 61) and light–dark box (LDB; between PD 62 and PD 63). For both tests, rearing and line crosses were scored in real-time, while remaining behaviours were scored later from video. Behavioural apparatuses were cleaned with 70% ethanol between rats and before the first rat was tested each day. The testing room was lit by a 60 W red light during both tests.

Behaviours measured included line crosses (between equal squares marked by white Fisherbrand^TM^ labelling tape (Thermo Fisher Scientific, Whitby, ON, Canada), total rearing, supported rearing, unsupported rearing, transitions between light and dark areas (in the LDB), time in centre (in the OFT; not including initial time taken to exit centre), time in light (in the LDB), latency to move from centre (in the OFT), and latency to move from the dark area (in the LDB). As described by Kalueff and Tuohimaa [42], increased time spent by a subject in (or transitions into) more anxiety-inducing areas (i.e., the centre area in the OFT; the light section in the LDB) was interpreted as showing less anxiety. Additionally, more time taken to leave a more-anxiety-inducing area when initially placed there (e.g., centre of OFT) is indicative of higher anxiety, whereas less time taken to voluntarily enter a more-anxiety-inducing area (e.g., light section of LDB when initially placed in the dark section) is indicative of lower anxiety [42]. Increased locomotion (e.g., line crosses) is usually regarded as a measure of lower anxiety in behavioural tests such as the OFT and LDB [42], but should be interpreted with caution when weight varies between tested groups [43]. Rearing frequency is another commonly examined behaviour, which may indicate more exploration (i.e., low anxiety) but can also vary due to overall locomotor ability, which is influenced by more than just anxiety (e.g., by weight) [44]. It has recently been suggested that rearing always be separated out by supported (locomotion measure to be interpreted cautiously) and unsupported (exploratory and low-anxiety behaviour), as these are distinct behavioural variables [45].

#### 2.5.1. Open Field Test

The OFT was constructed of black plexiglass (80 cm long × 80 cm wide × 35 cm high) with no lid (Figure 2B). Animals were initially placed in the centre of the arena [46,47], facing a consistent side of the testing apparatus.

#### 2.5.2. Light–Dark Box

The LDB (Figure 2C; overall 60 cm long × 30 cm wide × 45 cm high, 6 cm × 6 cm middle passage door; adapted from designs in [48,49]) had a 50/50 light/dark split where the dark box was constructed to fit into half of an overall apparatus constructed of clear plexiglass. The dark box was constructed of clear plexiglass on one side (to allow for video and live scoring) and opaque black plexiglass on the remaining three sides and hinged lid. At the start of testing, all rats were placed in the centre of the dark side, facing the opening to the light side. A 60 W white light was mounted 45 cm above the light section of the LDB, which was illuminated during testing.

### 2.6. Predator Odour Exposure, Sacrifice, and Tissue Collection

Between PD 71 and PD 74, animals were sacrificed 90 min following a 5-min acute predator odour stressor comprised of exposure to a cotton swab containing 0.5 mL of pure cat urine in a metal tea strainer hung from the side of the apparatus in the light section of the LDB (Figure 2C). This modified LDB was cleaned with 70% ethanol between each exposure and before the first exposure of the day. All surgical equipment was cleaned with 70% ethanol before the first sacrifice of the day and between animals. Testing, sacrifices, and dissections from the placebo group were always performed prior to the probiotic group.

All 62 rats were anaesthetised with Euthanyl (sodium pentobarbital) via intraperitoneal injection and, once fully unconscious, were quickly decapitated. Brains were removed and immediately placed on a plexiglass plate over dry ice, and the hippocampus and hypothalamus were gross dissected from a coronal section. Adrenal glands, colon, caecum, small intestine, stomach, fat, liver, and spleen were collected and immediately placed on dry ice until they were stored at −80 °C. The presence or absence of both *L. helveticus* R0052 and *B. longum* R0175 in all animals was confirmed in caecum contents collected at sacrifice, as previously described [37]. Briefly, we confirmed by qPCR that, on the day caecum was collected, CEREBIOME^®^ (previously known as Probio’Stick^®^) was detected in all probiotic-treated animals and not detected in any placebo-treated animals [37]. 

### 2.7. Plasma Inflammatory Marker Multiplex Analysis

For the detection of immunological analyte concentration in plasma (pg/mL), the Luminex Bio-Plex^®^ 200 system—along with the Bio-Plex Pro™ Rat Cytokine 23-Plex Assay Kit—was used (Bio-Rad Laboratories, Hercules, CA, USA, cat. #12005641, lot #64178837). Plasma was prepared from trunk blood collection after decapitation. At 4 °C, whole blood was spun at 1,000 *g* for 15 min, transferred into a clean microtube, re-spun at 10,000 *g* for 10 min, followed by transfer to a clean microtube. Plasma dilutions were done using the Bio-Plex^®^ sample diluent (part of the Bio-Plex Pro™ Rat Cytokine 23-Plex Assay Kit) at a 1:10 dilution, based on previous dilution optimisation. Following the manufacturer’s protocol, plasma samples were analysed using the Luminex Bio-Plex^®^ 200. Briefly, this kit is designed to measure the following pro-inflammatory, anti-inflammatory, and regulatory inflammatory analytes: IL (interleukin)-1α, IL-1β, IL-2, IL-4, IL-5, IL-6, IL-7, IL-10, IL-12p70, IL-13, IL-17A, IL-18, IFN-γ (interferon-gamma), M-CSF (macrophage-colony stimulating factor, CSF-1), GM-CSF (granulocyte macrophage-colony stimulating factor, CSF-2), G-CSF (granulocyte-colony stimulating factor, CSF-3), TNF-α (tumor necrosis factor-alpha), GRO/KC (growth related oncogene/keratinocyte chemoattractant, CXCL1, GRO-α), VEGF (vascular endothelial growth factor), MCP-1 (monocyte chemoattractant protein-1, CCL2), MIP-1α (macrophage inflammatory protein 1-alpha; CCL3), MIP-3α (CCL20), and RANTES (regulated upon activation normal T-cell expressed and secreted; CCL5).

### 2.8. Plasma Leptin Enzyme-Linked Immunosorbent Assay (ELISA)

For the detection of leptin concentration (pg/mL) in plasma, samples were previously prepared, as described in Section 2.7. Two leptin rat ELISA kits (ThermoFisher Scientific, Waltham, MA, USA, cat. #KRC2281, lot #218111-003) were counterbalanced by group type and for location on the plate. Samples were assayed in duplicate and diluted to 1:11 based on previous dilution optimisation. It was decided that samples with greater than 20% coefficient of variation (CV) would be redone, but no samples were assayed with greater than 20% CV. Interassay and intraassay variability are reported by the manufacturer as 4.6% and 3.6%, respectively.

### 2.9. Statistical Analyses

All statistical analyses were conducted using IBM SPSS Statistics (Version 25; IBM Corporation, Armonk, NY, USA) software. For behavioural analyses, outliers were removed from analyses if they were greater or below three standard deviations (*SD*) from the mean (*M*). There were no outliers found for adult weight or food intake variables. For plasma leptin levels, one extreme value just greater than 3 *SD*s from the mean (a placebo male exposed to Western diet) was noted, but the value remained in analyses because it represented a possible biological value on the standard curve with a CV of 0.87% between duplicates. For inflammation, outliers were removed from analyses if values were greater or less than 3 *SD*s from the mean for each analyte individually. Values below the standard curve were reported as the minimum for the standard curve for each analyte. Of the 23 analytes tested, only those deemed measurable were reported, with measurable being defined as 80% or greater of the subjects tested falling within the standard curve for that analyte.

All behavioural, adult weight, food intake, leptin, and inflammation dependent variables were analysed using 2 × 2 × 2 (treatment by diet by sex) factorial analyses of variance (ANOVAs). Results from all ANOVA analyses are presented with descriptive statistics (i.e., *M* and *SD*) provided for all measures, with the goal of conveying the amount of variability of data from the mean. Wean weight was analysed using a 2 × 2 (treatment by sex) factorial ANOVA, as offspring were not exposed to diet until after weaning. Further 2 × 2 × 2 ANOVAs requiring a co-variate (ANCOVAs) were conducted on all behavioural measures (controlling for adult weight). All 2-way and 3-way interactions were analysed post hoc by simple effects analyses. While the alpha level was chosen to be 0.05 and main effects and interactions are presented when results are below this threshold, due to the large number of ANOVAs being conducted in this study, it should be noted that *p*-values between 0.01 and 0.05 should be interpreted with caution as the possibility of type I error is increased with multiple comparisons. Correlations were analysed by Spearman’s rho (r_s_) also at an alpha level of 0.05 with *p*-values between 0.01 and 0.05 also interpreted with caution. Effect sizes were reported as partial eta squared (η_p_^2^), see [50,51] for considerations when using η_p_^2^ for power analyses and meta-analyses.

## 3. Results

### 3.1. Anxiety-Like Behaviours

A series of treatment by diet by sex factorial ANOVAs were conducted to analyse the six behavioural variables recorded from the OFT. Overall, there were no main effects of diet on any of these behavioural measures, but there were specific main effects of treatment and sex. First, for total rearing, a main effect of treatment was found (*p* = 0.002), in that placebo rats reared more overall compared to probiotic rats (Table 2). Specifically, there was a main effect of treatment for supported rearing (*p* < 0.001), whereby placebo rats performed more supported rears than probiotic rats (Table 2). No main effects of treatment were found for line crosses, unsupported rearing, time in centre, or latency from centre. Next, a main effect of sex was found for line crosses (*p* < 0.001), in that females crossed more lines than males (Table 2). Two main effects of sex were found for total rearing, in that females showed more overall rearing (*p* = 0.046) and supported rearing (*p* = 0.041) than males (Table 2). No sex differences were found for unsupported rearing, time in centre, or latency from centre.

Another series of treatment by diet by sex factorial ANOVAs were conducted to analyse the seven behavioural variables recorded from the LDB. Unsupported rearing in the LDB was not reported because the behaviour was too infrequently performed, but it is included in total rearing. Similar to OFT results, there were no main effects of diet on any of these behavioural measures, but there were main effects for treatment and sex, along with an interaction effect for the supported rearing behaviour. First, a main effect of treatment was found for supported rearing (*p* = 0.013), in that placebo rats engaged in more supported rears than probiotic rats and a main effect of treatment for transitions (*p* = 0.002) was found in that probiotic rats transitioned more between the light and dark compartments compared to placebo rats (Table 3). There were no main effects of treatment for line crosses, total rearing, time in light, or latency to light. Next, main effects of sex were found for line crosses (*p* < 0.001), supported rearing (*p* = 0.003), transitions (*p* < 0.001), and latency to light (*p* < 0.001), whereby females demonstrated more line crosses, more supported rearing, more transitions, and took less time to enter the light compartment of the LDB (Table 3). There were no main effects of sex for total rearing or time in light.

**Figure 3:**
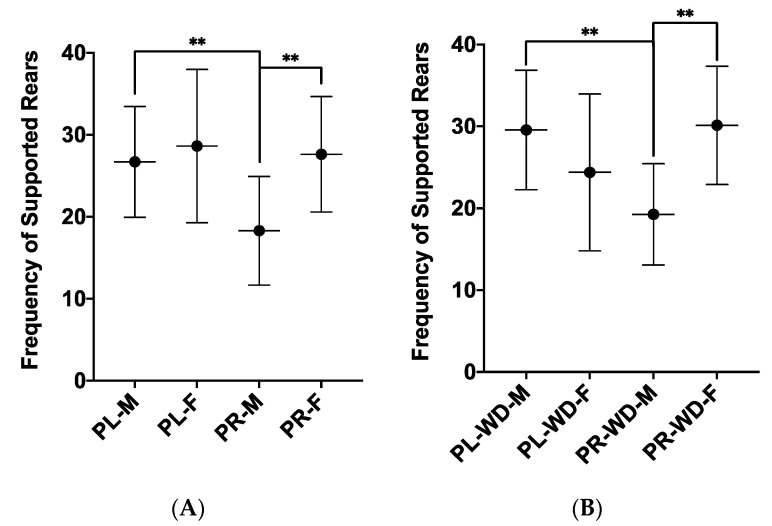
Supported rearing in the light–dark box for (**A**) the 2-way interaction between treatment and sex, and (**B**) the 3-way interaction between treatment, diet, and sex. Data expressed as *M*(*SD*). ** *p* < 0.01. PL: placebo; PR: probiotic; WD: Western diet; M: male; F: female.

ANOVA analyses from LDB data revealed a treatment by sex interaction for supported rearing (*F*_1, 54_ = 4.10, *p* = 0.048, *η*_p_^2^ = 0.070). Simple effects analyses showed that placebo males performed more supported rears compared to probiotic males (*p* = 0.002; Figure 3A). Furthermore, the probiotic females performed more supported rears than the probiotic males (*p* = 0.001; Figure 3A). A further treatment by diet by sex interaction was found for supported rearing (*F*_1, 54_ = 5.62, *p* = 0.021, η*_p_^2^* = 0.094), and simple effects analyses showed that placebo Western males performed more supported rears compared to probiotic Western males (*p* = 0.008; Figure 3B). As well, probiotic Western females performed more supported rears compared to probiotic Western males (*p* = 0.004; Figure 3B). With control diet females, those given the placebo reared more with support than those given the probiotic (*p* = 0.036), and with placebo females, those given the control diet reared more with support compared to those given the Western diet (*p* = 0.022). Additionally, females reared more than males in both placebo control animals (*p* = 0.019) and probiotic control animals (*p* = 0.036).

### 3.2. Metabolic Measures

#### 3.2.1. Wean and Adult Weights

**Figure 4:**
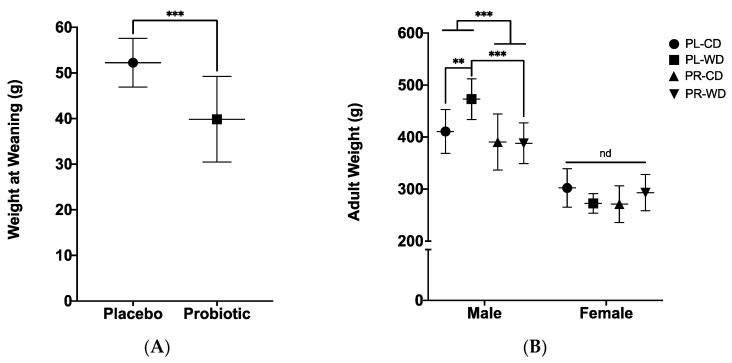
(**A**) Wean weight in placebo and probiotic animals; (**B**) Adult weight in all animals at an average of 67 days old. Data expressed as *M*(*SD*). ** *p* < 0.01, *** *p* < 0.001, nd: no difference. PL: placebo; PR: probiotic; WD: Western diet; CD: control diet.

A treatment by sex factorial ANOVA showed that wean weight was lower in offspring from mothers consuming probiotic during lactation (treatment main effect; *F*_1, 58_ = 40.36, *p* < 0.001, *η*_p_^2^ = 0.410; Figure 4A), with no difference in weight by sex found at weaning (*p* = 0.196). At birth, this difference was not apparent, with pups live-born to placebo-treated dams (*M* = 6.25, *SD* = 0.48, *n* = 33) having similar average weight to pups live-born to probiotic-treated dams (*M* = 6.26, *SD* = 0.51, *n* = 46). A treatment by diet by sex factorial ANOVA on adult weight found a main effect of sex (*F*_1, 54_ = 178.10, *p* < 0.001, *η*_p_^2^ = 0.767), in that males (*M* = 413.95, *SD* = 54.24) weighed more than females (*M* = 284.82, *SD* = 33.54). Furthermore, similar to wean weight, there was a main effect of treatment (*F*_1, 54_ = 8.66, *p* = 0.005, *η*_p_^2^ = 0.138), in that placebo rats (*M* = 359.54, *SD* = 88.55) had increased adult weight compared to probiotic rats (*M* = 335.83, *SD* = 67.75).

From the treatment by diet by sex factorial ANOVA on adult weight, a sex by treatment interaction was found (*F*_1, 54_ = 5.83, *p* = 0.019, *η*_p_^2^ = 0.097). Simple effects analyses showed that males in the placebo group weighed more in adulthood compared to males in the probiotic group (*p* < 0.001; Figure 4B), whereas no difference by treatment or diet was seen with female rats. A sex by diet by treatment interaction was also found (*F*_1, 54_ = 8.85, *p* = 0.004, *η*_p_^2^ = 0.141), where male rats in the Western diet condition weighed more in the placebo group than in the probiotic group (*p* < 0.001; Figure 4B). Furthermore, analyses of this three-way interaction showed that of males in the placebo group, those in the Western diet condition weighed more than males in the control diet condition (*p* = 0.004; Figure 4B).

#### 3.2.2. Average Daily Calorie Intake

Food intake was measured as average amount of calories consumed, calculated from grams of food eaten (see Section 2.4 above). It is noteworthy that adult weight was positively correlated with daily calorie intake (*r_s_* = 0.87, *p* < 0.001, *N* = 62). A treatment by diet by sex factorial ANOVA revealed a main effect of diet (*F*_1, 54_ *=* 16.47, *p* < 0.001, *η*_p_^2^ = 0.234), with Western diet animals (*M* = 113.72, *SD* = 35.87) consuming more calories compared to control diet animals (*M* = 100.94, *SD* = 22.42). There was also a main effect of sex (*F*_1, 54_ *=* 213.53, *p* < 0.001, *η*_p_^2^ = 0.798), in that males (*M* = 132.38, *SD* = 20.29) had increased calorie intake compared to females (*M* = 83.84, *SD* = 15.85). 

**Figure 5:**
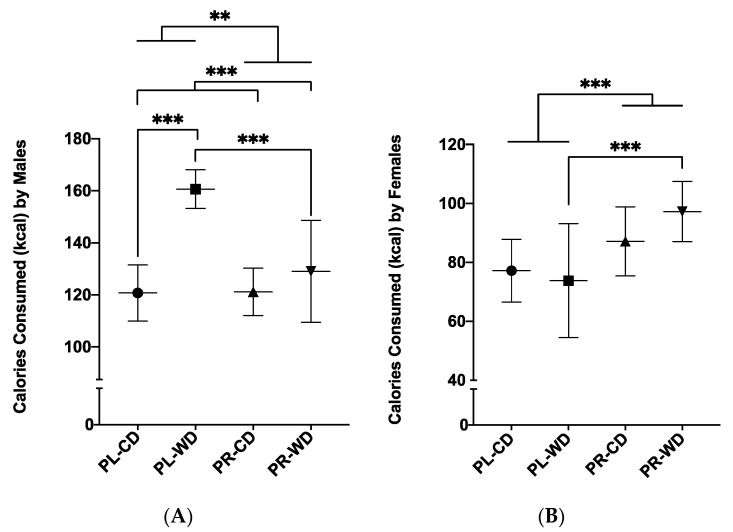
Food intake as measured by average calories consumed (kcal) over 4 days in placebo and probiotic rats given control diet or Western diet separated by (**A**) males and (**B**) females. Data expressed as *M*(*SD*). ** *p* < 0.01, *** *p* < 0.001. PL: placebo; PR: probiotic; WD: Western diet; CD: control diet.

ANOVA further revealed treatment by sex (*F*_1, 54_ *=* 23.08, *p* < 0.001, *η*_p_^2^ = 0.299) and diet by sex (*F*_1, 54_ *=* 9.37, *p* = 0.003, *η*_p_^2^ = 0.148) interactions for average daily calories consumed. Upon analysis of the treatment by sex interaction, it was first found that males in the placebo group consumed more daily calories compared to males in the probiotic group (*p* = 0.002; Figure 5A). On the contrary, probiotic females consumed more calories compared to placebo females (*p* < 0.001; Figure 5B). Analysis of the two-way diet by sex interaction also showed that in males only, animals given the Western diet consumed more calories compared to control diet animals (*p* < 0.001; Figure 5A).

A treatment by diet by sex interaction was found for daily calories consumed (*F*_1, 54_ *=* 11.44, *p* = 0.001, *η*_p_^2^ = 0.175) and simple effects analyses showed that in Western-diet-exposed males, placebo-treated animals consumed more calories compared to probiotic-treated animals (*p* < 0.001, Figure 5A). Additionally, within placebo-exposed males, Western diet rats consumed more calories than control diet rats (*p* < 0.001, Figure 5A). In females given the Western diet, probiotic animals consumed more calories compared to placebo animals (*p* < 0.001; Figure 5B).

#### 3.2.3. Plasma Leptin

**Figure 6:**
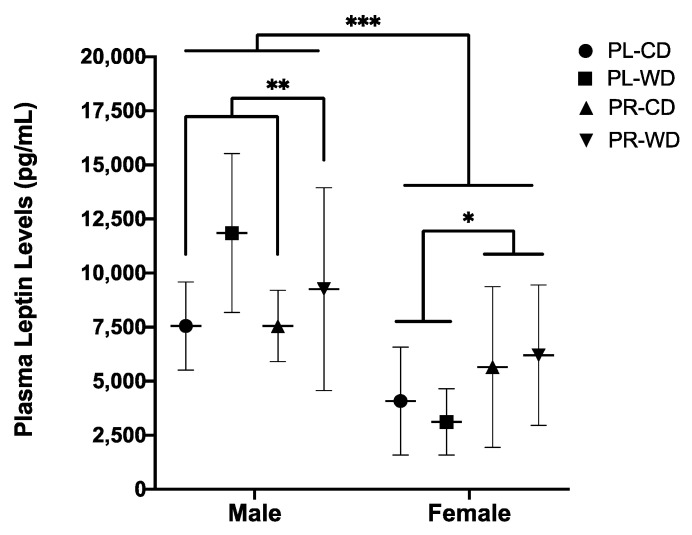
Plasma leptin levels (pg/mL) in males and females by treatment and diet condition. Data expressed as *M*(*SD*). * *p* < 0.05, ** *p* < 0.01, *** *p* < 0.001. PL: placebo; PR: probiotic; WD: Western diet; CD: control diet.

For leptin concentration in plasma, while the treatment by diet by sex factorial ANOVA did not reveal main effects of treatment or diet, there was a main effect of sex (*F*_1, 54_ = 30.11, *p* < 0.001, *η*_p_^2^ = 0.358), in that males (*M* = 9009.57, *SD* = 3560.35) had higher leptin levels at sacrifice compared to females (*M* = 4764.66, *SD* = 3000.57). Furthermore, there were treatment by sex (*F*_1, 54_ = 5.39, *p* = 0.024, *η*_p_^2^ = 0.091) and diet by sex (*F*_1, 54_ = 4.23, *p* = 0.045, *η*_p_^2^ = 0.073) interactions found. Simple effects analyses revealed that probiotic females had higher leptin levels than placebo females (*p* = 0.036; Figure 6). Simple effects analyses further revealed that males in the Western diet group had higher leptin levels than males in the control diet group (*p* = 0.010; Figure 6).

**Figure 7:**
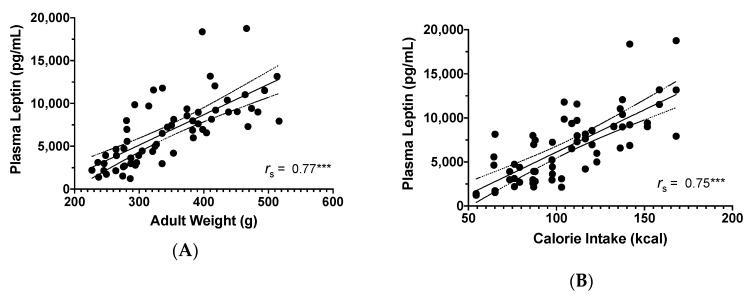
Scatterplot of the relationship between plasma leptin levels (pg/mL) and: (**A**) Adult weight (g); (**B**) Average daily calorie intake (kcal). *** *p* < 0.001, including 95% confidence bands of the best fit lines.

Plasma leptin levels were positively correlated with adult weight (*r_s_* = 0.77, *p* < 0.001, *N* = 62; Figure 7A) and daily calorie intake (*r_s_* = 0.75, *p* < 0.001, *N* = 62; Figure 7B).

### 3.3. Adult Weight and Behavioural Findings

**Figure 8:**
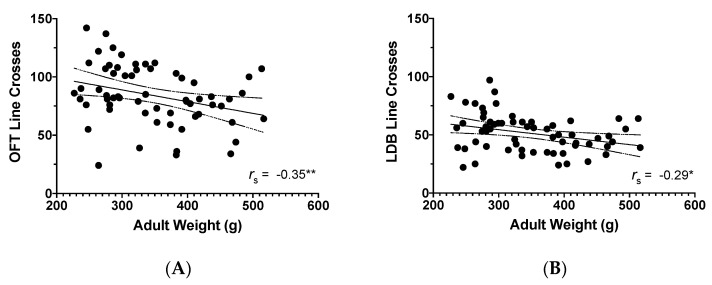
Scatterplot of the relationship between adult weight (g) and: (**A**) Line crosses in the open field test; (**B**) Line crosses in the light–dark box. *p < 0.05, **p < 0.01, including 95% confidence bands of the best fit lines.

Overall, lower adult weight was related to more locomotion (line crosses) in the OFT (*r_s_* = −0.35, *p* = 0.006, *N* = 62; Figure 8A). Adult weight was not correlated with any other measured variables in the OFT. Examining the correlations by treatment revealed a negative correlation between OFT line crosses and adult weight in placebo animals (*r_s_* = −0.39, *p* = 0.031, *n* = 30), but not in probiotic animals (*r_s_* = −0.25, *p* = 0.177, *n* = 32). Weight and line crosses were not correlated in males only (*r_s_* = 0.12, *p* = 0.525, *n* = 30) or in females only (*r_s_* = 0.11, *p* = 0.541, *n* = 32).

Due to main effects and interaction effects of treatment, diet, and sex on adult weight, along with correlations between weight and certain behaviours, a series of ANCOVAs on all dependent behavioural variables in the OFT were conducted. The main effects of treatment for total rearing (*F*_1, 53_ = 8.53, *p* = 0.005, *η*_p_^2^ = 0.139) and supported rearing (*F*_1, 53_ = 12.73, *p* < 0.001, *η*_p_^2^ = 0.197) remained, with placebo-treated rats rearing more overall and performing more supported rears than probiotic-treated rats. Females still performed more line crosses than males (sex main effect; *F*_1, 53_ = 7.32, *p* = 0.009, *η*_p_^2^ = 0.121), but females no longer performed more total rears (*F*_1, 53_ = 1.84, *p* = 0.180, *η*_p_^2^ = 0.034) or supported rears compared to males (*F*_1, 53_ = 2.85, *p* = 0.098, *η*_p_^2^ = 0.052). When controlling for adult weight, two main effects of sex appeared for time in centre (*F*_1, 51_ = 4.74, *p* = 0.034, *η*_p_^2^ = 0.085) and latency from centre (*F*_1, 51_ = 4.63, *p* = 0.036, *η*_p_^2^ = 0.083). Specifically, males (*M* = 12.64, *SD* = 13.62) took longer to leave the centre compared to females (*M* = 10.91, *SD* = 14.58) and males (*M* = 27.73, *SD* = 19.50) spent more time in the centre compared to females (*M* = 24.67, *SD* = 13.61).

**Figure 9:**
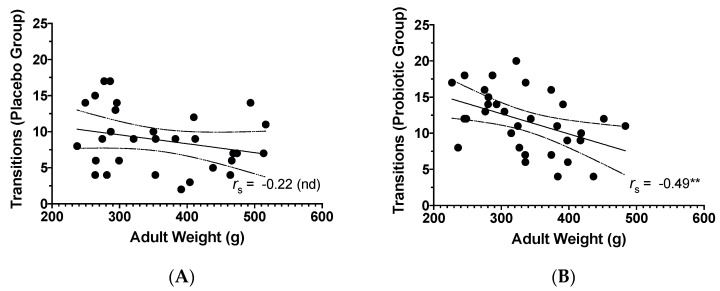
Scatterplot of the relationship between adult weight (g) and: (**A**) Transitions during light–dark box testing in placebo animals; (**B**) Transitions during light–dark box testing in probiotic animals. ** *p* < 0.01; nd: no difference; including 95% confidence bands of the best fit lines.

In the LDB, similar to the OFT, there was a negative correlation between line crosses and adult weight (*r_s_* = −0.29, *p* = 0.020, *N* = 62; Figure 8B) that was no longer present when examining the correlations by treatment group or by sex. Transitions in the LDB were higher in animals that weighed less (*r_s_* = −0.38, *p* = 0.002, *N* = 62). The negative correlation between transitions and adult weight was not present when only examining placebo animals (*r_s_* = −0.22, *p* = 0.243, *n* = 30; Figure 9A) or by examining results by sex, but was present with probiotic animals (*r_s_* = −0.49, *p* = 0.005, *n* = 32; Figure 9B). As well, overall, heavier animals in adulthood took longer to enter the light area (*r_s_* = 0.43, *p* < 0.001, *n* = 61). There was no correlation between latency to light and weight in placebo rats only (*r_s_* = 0.37, *p* = 0.052, *n* = 29) or by examining results by sex, but there was a positive relationship in probiotic rats (*r_s_* = 0.48, *p* = 0.006, *n* = 32). There were no other correlations for the remaining LDB behaviours and weight found, but upon examining all variables broken down by sex, a positive association was found in males between supported rearing and adult weight (*r_s_* = 0.52, *p* = 0.003, *n* = 30), showing that more supported rears were related to higher weight in males only.

ANCOVAs were also conducted for all LDB behavioural measures to examine differences in these behaviours after controlling for adult weight. Results showed that there was no longer a main effect of treatment on supported rearing (*F*_1, 53_ = 3.72, *p* = 0.059, *η*_p_^2^ = 0.066), but the main effect of treatment on transitions remained (*F*_1, 53_ = 9.90, *p* = 0.003, *η*_p_^2^ = 0.157), with probiotic rats transitioning more than placebo rats. Females still performed more line crosses (*F*_1, 53_ = 8.19, *p* = 0.006, *η*_p_^2^ = 0.134), along with more supported rears (*F*_1, 53_ = 6.73, *p* = 0.012, *η*_p_^2^ = 0.113) and transitions (*F*_1, 53_ = 7.01, *p* = 0.011, *η*_p_^2^ = 0.117). There was no longer a difference between males and females for latency to light (*F*_1, 52_ = 3.51, *p* = 0.066, *η*_p_^2^ = 0.063), no longer a two-way treatment by sex interaction for supported rearing (*F*_1, 53_ = 2.37, *p* = 0.130, *η*_p_^2^ = 0.043), or a three-way interaction for supported rearing (*F*_1, 53_ = 3.01, *p* = 0.089, *η*_p_^2^ = 0.054) after controlling for adult weight.

### 3.4. Plasma Inflammatory Analytes

Using multiplex technology, we analysed plasma samples from rats exposed to predator odour stress for the inflammatory analytes previously outlined in Section 2.7. The following 10 analytes were not consistently measured by the Bio-Plex Pro™ Rat Cytokine 23-Plex in rat plasma, and were thus excluded from analyses: IL-2, IL-4, IL-5, IL-6, IL-12p70, IL-13, IL-17A, IFN-γ, G-CSF, and TNF-α. The remaining 13 analytes that were deemed measurable included IL-1α (pro-inflammatory) [52,53,54], IL-1β (pro-inflammatory) [52,53,54,55], IL-7 (anti-inflammatory) [56], IL-10 (anti-inflammatory) [53,54,56,57,58], IL-18 (pro-inflammatory) [52,53,56], GM-CSF (pro-inflammatory) [53,59], GRO/KC (pro-inflammatory) [52], M-CSF (regulatory) [59], MIP-1α (anti-inflammatory) [56], MIP-3α (regulatory) [60], RANTES (pro-inflammatory) [52,53], VEGF (pro-inflammatory) [52], and MCP-1 (pro-inflammatory) [53,57].

ANOVA revealed that animals given the probiotic were higher in IL-1α (*p* = 0.022), IL-10 (*p* = 0.004), M-CSF (*p* < 0.001), and MIP-3α (*p* < 0.001; Table 4). ANOVA further revealed that the Western diet group was higher in IL-1β (*p* = 0.001), IL-7 (*p* < 0.001), GM-CSF (*p* < 0.001), GRO/KC (*p* = 0.018), MIP-1α (*p* = 0.006), VEGF (*p* = 0.019), and MCP-1 (*p* = 0.006; Table 5). By sex, males were higher in IL-1β (*p* = 0.037), IL-7 (*p* = 0.027), GM-CSF (*p* = 0.022), and MCP-1 (*p* = 0.003; Table 6). Overall, there were no main effects or interactions by treatment, diet, or sex for plasma levels of IL-18 and RANTES.

**Figure 10:**
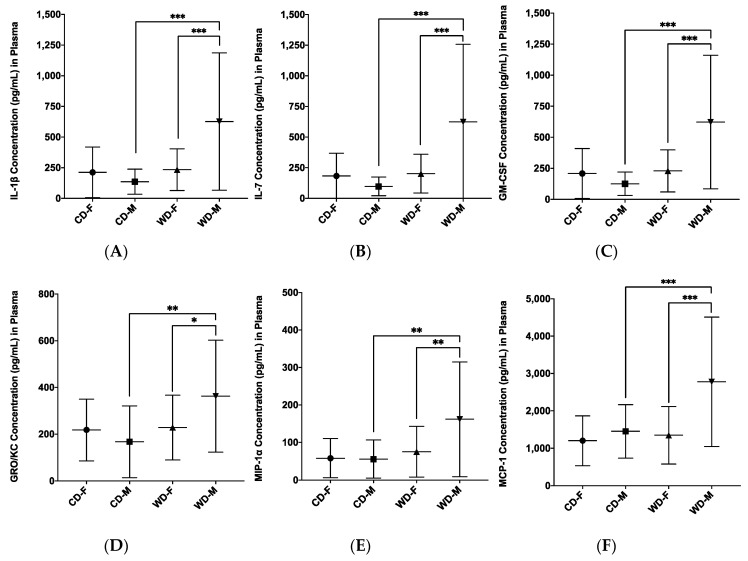
Diet by sex interactions in plasma inflammatory analytes from factorial ANOVA analyses for (**A**) IL-1β, (**B**) IL-7, (**C**) GM-CSF, (**D**) GRO/KC, (**E**) MIP-1α, and (**F**) MCP-1. Data expressed as *M*(*SD).* * *p* < 0.05, ** *p* < 0.01, *** *p* < 0.001. WD: Western diet; CD: control diet; M: male; F: female.

Diet by sex interactions were found (Figure 10) for IL-1β (*F*_1, 53_ = 10.13, *p* = 0.002, η_p_^2^ = 0.161), IL-7 (*F*_1, 53_ = 11.29, *p* = 0.001, η_p_^2^ = 0.176), GM-CSF (*F*_1, 53_ = 12.83, *p* < 0.001, η_p_^2^ = 0.195), GRO/KC (*F*_1, 53_ = 4.88, *p* = 0.031, η_p_^2^ = 0.084), MIP-1α (*F*_1, 53_ = 4.32, *p* = 0.042, η_p_^2^ = 0.075), and MCP-1 (*F*_1, 53_ = 5.28, *p* = 0.026, η_p_^2^ = 0.091). All diet by sex interactions were analysed post hoc by simple effects analyses, and two patterns again emerged. First, compared to male control rats, male Western diet rats were higher in IL-1β (*p* < 0.001), IL-7 (*p* < 0.001), GM-CSF (*p* < 0.001), GRO/KC (*p* = 0.002), MIP-1α (*p* = 0.001), and MCP-1 (*p* < 0.001). Second, compared to Western diet females, Western diet males were higher in IL-1β (*p* < 0.001), IL-7 (*p* < 0.001), GM-CSF (*p* < 0.001), GRO/KC (*p* = 0.032), MIP-1α (*p* = 0.007), and MCP-1 (*p* < 0.001).

**Figure 11:**
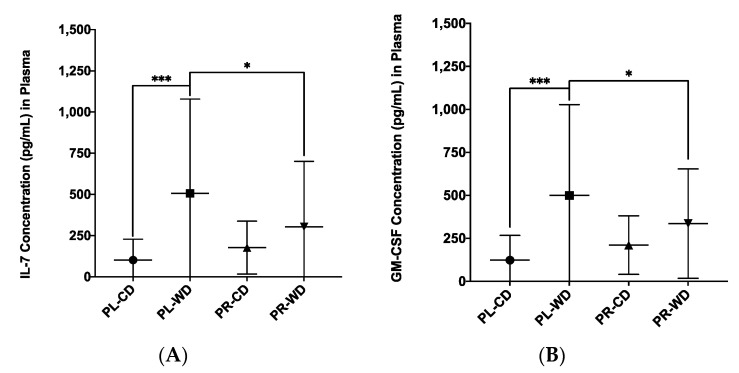
Treatment by diet interactions in plasma inflammatory analytes from factorial ANOVA analyses for (**A**) IL-7 and (**B**) GM-CSF. Data expressed as *M*(*SD*). * *p* < 0.05, *** *p* < 0.001. PL: placebo; PR: probiotic; WD: Western diet; CD: control diet.

Two treatment by diet interactions were found (Figure 11) for IL-7 (*F*_1, 53_ = 4.17, *p* = 0.046, η_p_^2^ = 0.073) and GM-CSF (*F*_1, 53_ = 4.48, *p* = 0.039, η_p_^2^ = 0.078). These treatment by diet interactions were analysed post hoc by simple effects analyses, and two patterns emerged. First, analyses revealed that compared to Western diet probiotic rats, Western diet placebo rats were higher in IL-7 (*p* = 0.034) and GM-CSF (*p* = 0.044). Second, compared to placebo control rats, placebo Western diet rats were higher in IL-7 (*p* < 0.001) and GM-CSF (*p* < 0.001).

## 4. Discussion

The goal of this work was to increase knowledge on the health effects of *L. helveticus* R0052 and *B. longum* R0175 (i.e., CEREBIOME^®^) using a developmental model. To summarise, two tests of anxiety, the OFT and LDB, were used in the present study with consistent results. After controlling for adult weight, placebo-treated rats showed evidence of higher anxiety [42,45,49,61]. Again, after factoring in weight, females showed evidence of lower anxiety [42]. This is the first animal study to our knowledge that has examined the metabolic consequences of CEREBIOME^®^, and our metabolic results suggest drastically different weight and food intake patterns in male and female rats with respect to probiotic treatment and diet. In females, treatment with the probiotic resulted in higher leptin levels compared to placebo and increased calorie intake, especially with Western diet exposure, without the expected change in weight. In males, the probiotic counteracted the increase in daily calories consumed and adult weight that was seen in the placebo-treated animals given the Western diet, with no corresponding change in leptin levels by treatment (i.e., leptin levels were higher only with Western diet exposure). It is also important to note that wean weight was lower in the offspring of dams given probiotic compared to placebo, even with no difference in birth weight, suggesting that the probiotic could be modulating weight during lactation either directly (i.e., the probiotic is being transferred to the pups through the breast milk) or indirectly (i.e., the pups are receiving probiotic from being in the same environment as their mother). Finally, approximately 90 min after rats were exposed to acute predator odour stress, specific inflammatory analytes, seemingly independent of analyte category, were higher in those given CEREBIOME^®^, in those treated with a Western diet, and in males.

The fact that placebo-treated rats reared more with support than probiotic-treated rats is noteworthy, as probiotic treatment is reported to improve anxiety in animal studies [18,24] and decrease the stress response [15,16,62,63]. Rearing is a complex behaviour (especially supported rearing) [45], and should be interpreted with caution as the act of performing a supported rear can vary based on the state of the animal performing the behaviour (i.e., escape behaviour or exploratory behaviour) [61]. From our results, it can only be theorised that the placebo animals may be performing more supported rears due to a desire to escape the testing area. This idea is strengthened by the fact that probiotic-treated animals transitioned more in the LDB (a behaviour consistent with lower anxiety) and reveals that multiple means for estimating anxiety levels is ideal. Future studies would benefit from additional measured variables such as number of defecations to help distinguish between exploratory- versus escape-related rearing [64].

From previous literature describing the effects of diet on the stress response system [27,65], it is of interest that we did not see any effect of diet on our anxiety measures. This is consistent with Abildgaard and colleagues [62], who also found no differences in the OFT by probiotic treatment or by diet, but they solely measured locomotion. It may be that results are affected by the type of diet, length of stress exposure, and choice of behavioural tests. One important note is that a high-carbohydrate control diet such as that used here leads to insulin resistance, excess fat storage, and weight gain [66], and low-carbohydrate diets are used when treating insulin resistance and type 2 diabetes [67]. High-carbohydrate diets are defined as containing greater than 45% of their energy from carbohydrates [67]—markedly lower than commonly used control diets, which are frequently comprised of 70% carbohydrates. In fact, Prasad and Prasad [68] report that rats given a 90% high-fat diet for one week had decreased observed anxiety on the elevated plus maze (EPM) test compared to baseline when compared to a 90% carbohydrate and 90% protein diet. Additionally, while Bridgewater et al. [46] found that male mice (but not females) given a 60% high-fat diet showed more anxiety-like behaviours in the OFT and EPM when also exposed to a chronic unpredictable stress paradigm. Importantly, the study by Bridgewater and colleagues [46] obtained similar results to the present study, in that male and female mice responded uniquely with respect to their observed anxiety.

*Lactobacillus* and *Bifidobacterium* strains have been documented as agents that can aid in the prevention and reversal of obesity via their impact on modulating the gut microbiome (i.e., affecting energy balance/storage, food intake hormones, and the lining of the gut) [69]. Previous research has shown that weight gain and other markers of obesity are improved with probiotic treatment (e.g., *Bifidobacterium* strains [20]; *L. helveticus* R0052 [22]). At least for males, our results suggest that probiotic treatment may lessen the negative health impacts of the Western diet. Our results are consistent with literature characterising the effects of probiotics on weight gain in response to unhealthy diet (e.g., in mice [22]; in zebrafish [70]). Although sex-specific effects of high-fat diet have been documented [46], there is a stark lack of studies examining how the combination of diet and probiotic treatment might affect males compared to females. For instance, Karlsson et al. [19] reported lower weight gain, fat around organs, and plasma leptin with *Lactiplantibacillus plantarum* treatment, but results were not reported by sex. In a recent study, individuals with major depressive disorder were supplemented with the CEREBIOME^®^ formulation, a galactooligosaccharide prebiotic, or placebo, and it was found that only the probiotic was associated with decreased reported depressive symptoms on the Beck Depression Inventory [71]. Of interest was that this probiotic was related to an increase in the ratio of tryptophan to isoleucine (a branched-chain amino acid, BCAA), with no change in overall tryptophan levels, suggesting that the probiotic may be related to decreased levels of isoleucine [71]. Lee et al. [72] recently found that overall BCAA levels in plasma (i.e., sum of valine, leucine, and isoleucine) was associated with metabolic dysfunction and diabetes, so further studies that aim to characterise the metabolic effects of CEREBIOME^®^ would benefit from including a measure of overall or specific BCAAs.

Because leptin is reported to inhibit food intake [73], it is of interest that the female rats given probiotic had both increased leptin and increased calorie intake compared to those given placebo. There is evidence of sex differences in gut microbiota compositions. For instance, Tennoune et al. [74] report *Escherichia coli* K12 DNA presence at baseline in female rat feces only. Interestingly, the group further reports increased weight in females after administration of this same strain but both decreased weight and decreased food intake in males [74]. As Taraschenko and colleagues [75] suggested, evidence shows that diet-induced obesity occurs differently in each sex, highlighting the need for studies to report on results in both sexes when examining the anti-obesity effects of probiotic treatment. One study that aimed to correct high-fat diet-induced obesity was only successful with male rats [75]. Furthermore, Alonso-Caraballo and colleagues [76] found that increased anxiety-like behaviours in the EPM were associated with increased weight gain and plasma leptin in male rats only. Interestingly, when Harris et al. [77] induced leptin resistance (by administering high-fat diet and leptin injections) females still lost weight in response to the leptin administration with no change in food intake (i.e., they did not become leptin resistant like males) [77]. The results from Harris et al. [77] combined with the present results may indicate a time-delay in the development of leptin resistance in female rats compared to male rats. As Gruzdeva et al. [78] describe in their review, increased leptin can reduce appetite and body weight to a certain extent, but if the body becomes resistant to leptin as seen with obesity, increased leptin signals are no longer effective in decreasing food intake. Although leptin resistance is difficult to characterise and the mechanisms underlying leptin resistance have not been fully elucidated [78], the male rats in the present study had greater plasma leptin levels compared to females, which may be indicative of leptin resistance. Thus, if females were not in a leptin resistance state, higher leptin levels in the probiotic-treated compared to the placebo-treated females may indicate a more metabolically healthy outcome at the time leptin was measured. 

Our results from inflammatory marker analysis are intriguing as we expected that probiotic treatment would both decrease pro-inflammatory markers and increase anti-inflammatory markers. In fact, probiotic animals displayed higher levels of pro-inflammatory analyte, IL-1α, but also of the anti-inflammatory analyte, IL-10, and the regulatory analytes, M-CSF and MIP-3α. Prior studies have shown that probiotic treatment decreases pro-inflammatory analytes and factors in response to chronic stress (e.g., IL-6 in rats [23]; IL-1β and NF-κB in Syrian golden hamsters [24]). Furthermore, Dai and colleagues [79] administered a probiotic including *Bifidobacteria*, *Lactobacilli*, and one *Streptococcus* species in a rat model of colitis and found that treatment similarly induced production of IL-10 (anti-inflammatory), but concurrently, reduced production of pro-inflammatory analytes (e.g., TNF-α and IL-6 in colon and serum). In addition, Bisson et al. [80] showed that a probiotic given to rats—containing *L. helveticus* R0052, *B. longum* R0175, *Lacticaseibacillus rhamnosus* R0011 and *Saccharomyces boulardii* yeasts—reduced pro-inflammatory analytes in serum (e.g., IL-1α, IL-6) and induced production of IL-4 and IL-10 after *Escherichia coli* infection. However, research is conflicted with respect to the effects of probiotic treatment on the inflammatory response, especially after stress exposure. In rats, Ait-Belgnaoui et al. [15] found no changes in plasma pro-inflammatory cytokines (i.e., IL-1β, IL-6) after probiotic (*Companilactobacillus farciminis*) treatment or acute stress exposure (partial restraint stress), but did find lower levels of hippocampal inflammatory analytes (IL-1β, IL-6, TNF-α mRNA) and stress hormones after stress exposure (i.e., CRF mRNA, plasma corticosterone and adrenocorticotropic hormone, ACTH) with the probiotic treatment. Conversely, in response to a multi-species probiotic treatment with prior stress exposure, Abilgaard et al. [62] found that mononuclear cells isolated from rat blood produced a greater amount of the cytokines IFN-γ, IL-2 and IL-4 when stimulated. As well, mice that were immune challenged and then exposed to an acute footshock stressor had increased glucocorticoid release and reduced IL-1α and IL-1β in the brain [81]. 

A complex relationship exists between the interleukin-1 family and the HPA axis where low levels of IL-1 (e.g., IL-1α and IL-1β) might be beneficial for efficient coping with stressors, but under severe or chronic stress conditions, increased IL-1 might be an adaptive method in reducing the stress response and preventing widespread damage [82]. In fact, one study that used the same combination of *L. helveticus* R0052 and *B. longum* R0175 as the present study found that, in rats, the probiotic prevented increased stress hormone release (i.e., corticosterone, noradrenaline, adrenaline) in plasma and prevented downregulation of glucocorticoid receptors, seen in response to chronic stress [16]. Wagner and Johnson [83] reported that cells from a vaginal epithelial cell line infected with *Candida albicans* and exposed to *Lacticaseibacillus rhamnosus* GR-1® and *Limosilactobacillus reuteri* RC-14® responded by increasing expression of IL-1α and IL-1β mRNA. Taken together, if the placebo animals had a greater physiological stress response to the potent predator odour stress (i.e., severe acute stress), this may explain the relatively lower amounts of plasma IL-1α measured in placebo compared to probiotic animals. Although research is severely lacking on the effects of stress and probiotic treatment on less-studied immunological analytes such as M-CSF and MIP-3α, it would not be surprising if these analytes are also affected by the stress response. 

Our findings that the Western diet-exposed animals had increased inflammatory marker release is interesting when considering the relationship between glucocorticoids (steroid hormones) and the immune response. Glucocorticoids, when administered therapeutically, can modulate the immune system in response to autoimmune diseases and organ transplantation but can also promote insulin resistance and diabetes development (see [84] for a commentary). Furthermore, Wang et al. [85] showed that animals on a high-sugar diet had increased plasma corticosterone levels. Taking into consideration the research reported above, increased carbohydrate intake from the control diet may have led to increased presence of stress hormones, which in turn, could help to explain a lower inflammatory response in our control diet group. 

We noted a number of sex differences in plasma analyte levels that may be explained, at least in part, by levels of circulating sex steroid hormones, and, is of importance to further studies characterising the effects of probiotic treatment in both sexes. Pyter et al. [86] reported that chronic stress exposure led to increased hippocampal inflammatory marker expression in male, but not female rats, after an immune challenge with lipopolysaccharide. Although there were no sex differences in corticosterone levels after the immune challenge, female rats had increased plasma estradiol, which may have prevented hippocampal expression of genes for the inflammatory analytes measured (e.g., IL-1β, TNF-α) [86]. Both testosterone (indirectly) and estradiol (directly) affect the HPA axis related to their actions on androgen and estrogen receptors, respectively [87] and males naturally have lower circulating levels of estradiol compared to females [88]. Furthermore, in response to stress, a recent review reports that female rats have increased corticosterone and ACTH compared to males [87], which may also suppress the inflammatory response as described above and may be further affected by circulating levels of estradiol. Theoretically, increased estrogens and/or increased corticosterone release may affect the inflammatory response in females and warrants investigation in studies aiming to elucidate sex differences in the mechanistic link between stress and inflammation.

## 5. Conclusions

We present evidence that the probiotic-treated animals showed less anxiety in the behavioural testing arenas used in this study (i.e., the OFT and LDB). Future studies would benefit from combining further anxiety behavioural tests (e.g., the EPM) with the aim of increasing the duration and complexity of testing in order to characterise the more subtle effects of probiotic treatment (e.g., to increase the incidence of lower-frequency behaviours). Importantly, by examining behavioural results by sex while factoring in weight, we show that weight is not the only factor affecting the differences in the observed anxiety-like behaviours seen between males and females. With the drastically different pattern of results between males and females seen for our metabolic measures (i.e., adult weight, food intake, and plasma leptin), it becomes important for future studies to include sex as a factor in the study design. This is the first study to our knowledge that examined inflammatory levels after acute exposure to predator odour stress with CEREBIOME^®^ treatment. These results provide evidence that male, probiotic-treated, and Western-diet-administered rats have a greater inflammatory response after acute stress, which may be related to increased levels of specific steroid hormones and warrants further study (e.g., estradiol in males, corticosterone in placebo-treated and control diet-fed animals).

Overall, this study adds to what we know about a combination *L. helveticus* R0052 and *B. longum* R0175 probiotic with respect to adult anxiety behaviours, weight, food intake, and plasma levels of leptin and inflammatory analytes in a developmental Long–Evans rat model also exposed to a control or Western diet. The examination of these factors in the same group of animals is important, as all of the abovementioned health outcomes have been shown to be affected by probiotic treatment and each other. Importantly, our results demonstrate specific benefits of this probiotic that vary by sex, which is a critical consideration for developmental animal studies, which often examine effects in males exclusively. Over and above the specific findings in this study, it is our hope that results demonstrate the importance of including both sexes in animal studies that examine health outcomes and call attention to the idea of appropriate caution when choosing a control diet for a high-fat or Western diet. In future studies examining the effects of Western diet exposure, it would be enlightening to compare to the rat chow traditionally provided by animal care facilities rather than a high-carbohydrate control diet.

## Figures and Tables

**Table 1:** Litter diversity details for each of the eight experimental groups including total sample size, maximum subjects per litter in each group, and represented litters of total by treatment (four probiotic, three placebo). PL: placebo; PR: probiotic; WD: Western diet; CD: control diet; M: male; F: female.

	Experimental Group							
	PR-WD-M	PR-WD-F	PR-CD-M	PR-CD-F	PL-WD-M	PL-WD-F	PL-CD-M	PL-CD-F
Sample size	8	8	8	8	7	8	7	8
Max. *n* per litter	3	3	2	3	5	4	3	3
Represented litters	4 of 4	3 of 4	4 of 4	3 of 4	2 of 3	2 of 3	3 of 3	3 of 3

**Table 2:** Overview of treatment and sex main effects in the open field test from factorial ANOVA analyses for line crosses, total rearing, and supported rearing, including means (*M*) and standard deviations (*SD*).

**Behaviour, *df***	**Probiotic** ***M*** **(*SD*)**	**Placebo** ***M* (*SD*)**	***F*-Value**	***p-*Value**	**Effect Size (η_p_^2^)**
Total Rearing, *F*_1, 54_	20.22 (8.69)	29.03 (11.85)	11.19	*p* = 0.002	*η*_p_^2^ = 0.172
Supported Rearing, *F*_1, 53_	15.81 (6.11)	24.07 (9.59)	17.21	*p* < 0.001	*η*_p_^2^ = 0.245
**Behaviour, *df***	**Male** ***M* (*SD*)**	**Female** ***M*** **(*SD*)**	***F*-Value**	***p-*Value**	**Effect Size (η_p_^2^)**
Line Crosses, *F*_1, 54_	72.73 (21.39)	94.69 (24.25)	14.06	*p* < 0.001	*η*_p_^2^ = 0.207
Total Rearing, *F*_1, 54_	21.73 (10.21)	27.31 (11.62)	4.17	*p* = 0.046	*η*_p_^2^ = 0.072
Supported Rearing, *F*_1, 53_	17.63 (8.19)	21.77 (9.24)	4.39	*p* = 0.041	*η*_p_^2^ = 0.076

**Table 3:** Overview of treatment and sex main effects in the light–dark box from factorial ANOVA analyses for line crosses, supported rearing, transitions, and latency to enter light, including means (*M*) and standard deviations (*SD*).

**Behaviour, *df***	**Probiotic** ***M*** **(*SD*)**	**Placebo** ***M* (*SD*)**	***F*-Value**	***p-*Value**	**Effect Size (η_p_^2^)**
Supported Rearing, *F*_1, 54_	22.97 (8.22)	27.73 (8.16)	6.61	*p* = 0.013	*η*_p_^2^ = 0.109
Transitions, *F*_1, 54_	11.69 (4.21)	8.83 (4.19)	10.21	*p* = 0.002	*η*_p_^2^ = 0.159
**Behaviour, *df***	**Male** ***M* (*SD*)**	**Female** ***M* (*SD*)**	***F*-Value**	***p-*Value**	**Effect Size (η_p_^2^)**
Line Crosses, *F*_1, 54_	43.30 (10.92)	58.97 (16.81)	18.63	*p* < 0.001	*η*_p_^2^ = 0.256
Supported Rearing, *F*_1, 54_	22.23 (7.84)	28.13 (8.15)	9.41	*p* = 0.003	*η*_p_^2^ = 0.148
Transitions, *F*_1, 54_	8.17 (3.53)	12.31 (4.24)	20.66	*p* < 0.001	*η*_p_^2^ = 0.277
Latency to Light, *F*_1, 53_	50.39 (44.06)	17.90 (26.37)	13.55	*p* < 0.001	*η*_p_^2^ = 0.204

**Table 4:** Overview of treatment main effects for plasma inflammatory analytes from factorial ANOVA analyses for IL-1α, IL-10, M-CSF, and MIP-3α, including means (*M*) and standard deviations (*SD*).

Analyte, *df*	Probiotic *M* (*SD*)	Placebo *M* (*SD*)	*F*-Value	*p*-Value	Effect Size (η_p_^2^)
IL-1α, *F*_1, 53_	163.79 (126.86)	94.81 (95.64)	5.54	*p* = 0.022	*η*_p_^2^ = 0.095
IL-10, *F*_1, 53_	100.15 (94.01)	37.04 (63.77)	8.95	*p* = 0.004	*η*_p_^2^ = 0.145
M-CSF, *F*_1, 53_	24.97 (11.51)	13.54 (11.22)	14.40	*p* < 0.001	*η*_p_^2^ = 0.214
MIP-3α, *F*_1, 51_	31.40 (11.49)	19.93 (6.53)	20.85	*p* < 0.001	*η*_p_^2^ = 0.290

**Table 5:** Overview of diet main effects for plasma inflammatory analytes from factorial ANOVA analyses for IL-1β, IL-7, GM-CSF, GRO/KC, MIP-1α, VEGF, and MCP-1, including means (*M*) and standard deviations (*SD*).

Analyte, *df*	Western *M* (*SD*)	Control *M* (*SD*)	*F*-Value	*p*-Value	Effect Size (η_p_^2^)
IL-1β, *F*_1, 53_	416.93 (441.58)	175.24 (166.16)	12.00	*p* = 0.001	*η*_p_^2^ = 0.185
IL-7, *F*_1, 53_	398.52 (488.85)	141.26 (147.92)	12.91	*p* < 0.001	*η*_p_^2^ = 0.196
GM-CSF, *F*_1, 53_	412.78 (429.38)	168.43 (161.61)	14.99	*p* < 0.001	*η*_p_^2^ = 0.220
GRO/KC, *F*_1, 53_	291.18 (200.57)	193.62 (142.54)	6.00	*p* = 0.018	*η*_p_^2^ = 0.102
MIP-1α, *F*_1, 53_	115.89 (121.43)	57.20 (50.43)	8.03	*p* = 0.006	*η*_p_^2^ = 0.132
VEGF, *F*_1, 52_	456.43 (383.96)	250.07 (265.76)	5.83	*p* = 0.019	*η*_p_^2^ = 0.101
MCP-1, *F*_1, 53_	2014.98 (1476.12)	1321.93 (689.83)	8.10	*p* = 0.006	*η*_p_^2^ = 0.133

**Table 6:** Overview of sex main effects for plasma inflammatory analytes from factorial ANOVA analyses for IL-1β, IL-7, GM-CSF, and MCP-1, including means (*M*) and standard deviations (*SD*).

Analyte, *df*	Male *M* (*SD*)	Female *M* (*SD*)	*F*-Value	*p*-Value	Effect Size (η_p_^2^)
IL-1β, *F*_1, 53_	372.48 (461.38)	223.07 (186.10)	4.60	*p* = 0.037	*η*_p_^2^ = 0.080
IL-7, *F*_1, 53_	351.46 (511.02)	191.94 (170.06)	5.16	*p* = 0.027	*η*_p_^2^ = 0.089
GM-CSF, *F*_1, 53_	365.50 (450.16)	218.91 (183.12)	5.59	*p* = 0.022	*η*_p_^2^ = 0.095
MCP-1, *F*_1, 53_	2091.35 (1451.34)	1274.37 (710.64)	9.83	*p* = 0.003	*η*_p_^2^ = 0.157
